# Water Diffusion through a Titanium Dioxide/Poly(Carbonate Urethane) Nanocomposite for Protecting Cultural Heritage: Interactions and Viscoelastic Behavior

**DOI:** 10.3390/nano7090271

**Published:** 2017-09-13

**Authors:** Mario Abbate, Loredana D’Orazio

**Affiliations:** Istituto per i Polimeri, Compositi e Biomateriali, Via Campi Flegrei, 34, Fabbricato 70, 80078 Pozzuoli (Naples), Italy; mario.abbate@ipcb.cnr.it

**Keywords:** Polymer/TiO_2_ nanocomposites, thermoplastic polyurethanes, diffusion barrier, sorption, cultural heritage

## Abstract

Water diffusion through a TiO_2_/poly (carbonate urethane) nanocomposite designed for the eco-sustainable protection of outdoor cultural heritage stonework was investigated. Water is recognized as a threat to heritage, hence the aim was to gather information on the amount of water uptake, as well as of species of water molecules absorbed within the polymer matrix. Gravimetric and vibrational spectroscopy measurements demonstrated that diffusion behavior of the nanocomposite/water system is Fickian, i.e., diffusivity is independent of concentration. The addition of only 1% of TiO_2_ nanoparticles strongly betters PU barrier properties and water-repellency requirement is imparted. Defensive action against penetration of water free from, and bonded through, H-bonding association arises from balance among TiO_2_ hydrophilicity, tortuosity effects and quality of nanoparticle dispersion and interfacial interactions. Further beneficial to antisoiling/antigraffiti action is that water-free fraction was found to be desorbed at a constant rate. In environmental conditions, under which weathering processes are most likely to occur, nanocomposite *Tg* values remain suitable for heritage treatments.

## 1. Introduction

Cultural heritage assets are exposed to weather and submitted to influence of environmental parameters in a world where the climate is changing. Physical, chemical, and biological factors interact with constitutive materials inducing changes both in their compositional and structural characteristics [1,2,3]. The great importance of water as a threat to heritage is acknowledged: in natural conditions atmospheric water is the main agent associated with stone degradation, acting mainly through capillary rising. Rainwater penetrating by absorption is a vehicle of airborne acidic pollutants interacting with stone through chemical reactions of dissolved CO_2_, NO_x_, and SO_2_. Moreover, water changes cohesion properties of the stone crystalline structure through physical/mechanical decay due to thermal excursions in wet conditions (freeze-thaw cycles) [4]. Hence, the need to improve effectiveness and eco-sustainability of preventive conservation and maintenance solutions are grown hugely.

Different classes of polymers have been so far employed as protective coatings of stone heritage without adequate knowledge of the properties of both plain polymer and polymer/substrate system [5,6,7]. As a result, insufficient efficacy and/or poor weatherability was usually observed. Such polymeric materials in most cases only provide short-term water repellency of the treated surfaces and are intrinsically unstable in photo-oxidative conditions typical of outdoor exposure. Notwithstanding that even polymers with partially fluorinated, side chains were ad hoc synthesized and tested to increase water repellency effectiveness and coating Ultraviolet (UV) radiation stability [8], presently the scientific community is still far from the achievement of materials fulfilling all the fundamental requirements of protective coatings [9].

While the last decade has seen several advancements in the field of polymer nanocomposites for a wide range of mechanical, electronic, magnetic, biological, and optical properties, fewer efforts have been focused on designing such a nanomaterial with optimal macroscale properties for protecting cultural heritage. A nanocomposite’s properties depend ultimately upon a myriad of variables that include the quality of dispersion, interfacial adhesion, extent of region between nanoparticles fillers and bulk polymer matrix, processing methods, loading of the particles, modification of the surfaces of nanoparticles, aspect ratio of particles, compatibility of particles and host polymer, size of particles, radius of gyration of the host polymer and the properties of the constituents. Even though in literature structure-property relationships are lacking, it is evident that the properties of polymer nanocomposites are highly sensitive to both the quality of dispersion and region between nanoparticles fillers and bulk polymer matrix and that small changes in processing conditions, particle size, or chemistry dramatically affects these two key factors [10].

Recently results were achieved by matching a polymer with proper end properties, including eco-sustainable usage and non-toxicity, to create an inorganic photocatalytic nanocompound that was efficient in de-soiling and had biocide activities. A polymeric coating for protecting cultural heritage based on a water-dispersed TiO_2_/poly (carbonate urethane) nanocomposite was prepared by a low impact procedure, i.e., cold mixing of the single components via sonication [11]. By means of the polymeric nanocomposites technology, highly innovative and outstanding performances were also achieved in terms of stability and durability as compared with other treatments based on acrylic and vinylic polymers widely used in conservation and restoration [6,7].

The next step of our investigation is concerned with applications of nanocomposite water dispersions on a porous degradable stone to demonstrate treatments’ aesthetical compatibility, and ability in reducing soiling and biocide properties [12]. For a given nanocomposite concentration (*w*/*v* %), water Absorption Coefficients (ACs) of untreated and treated stone samples were also evaluated according to NORmalizzazione MAteriali Lapidei (NORMAL) 11/85 [13] as a function of the application procedure; i.e., air-brush until the stone surface was saturated, following a widespread practice in conservation, and full immersion in nanocomposite dispersions at room temperature for 1 h. The AC values achieved, pertaining to stone characterization, indicated that the treatments performed slowed the rate of water absorption of the stone. Hence, the nanocomposite homogeneous, transparent, colorless film formed by water casting at room temperature was proved to protect stone against water penetration.

The present work is focused, conversely, on water diffusion characteristics through TiO_2_/poly (carbonate urethane) nanocomposite film samples, the novelty consisting of an in-depth analysis, on one hand, of nanocomposite diffusivity, and of the other hand, of effects of water uptake amount and nanoparticles/matrix interactions on glass transition temperature (*Tg*) of Polyurethane (PU) soft and hard domains. As a matter of fact, *Tg* has a deep influence on transport properties and, for applications of polymer-based materials in the field of cultural heritage, *Tg* is, as well, a relevant requirement. Coatings with a *Tg* value considerably higher than room temperature cannot be able to react to dimensional changes of treated items, whereas coatings with *Tg* values conspicuously lower than room temperature are much too soft for working and moreover are inclined to pick up dirt. Nanocomposite water diffusion coefficients were determined by means of gravimetric techniques combined with on time-resolved Fourier Transform (FT)-Near Infrared (NIR) measurements and compared to that exhibited by pristine PU matrix. Moreover, vibrational spectroscopy was selected as one of the best-suited techniques for probing hydrogen-bonded molecular structures [14] with the aim of gathering information on amount of water uptake, as well as, of species of water molecules absorbed within polymer matrix in presence of TiO_2_ nanoparticles. In particular, significant effects of the addition of 1% of TiO_2_ nanoparticles on amount of water free from, and strongly bonded through, H-bonding association absorbed/desorbed within the PU matrix, at environmental conditions under which weathering processes are most likely to occur, were highlighted. Correlations between adsorbed water amount and nanocomposite viscoelastic behavior were also established through Dynamic Mechanical Thermal Analysis (DMTA). 

## 2. Results

### 2.1. Gravimetric Measurements 

Because water absorption of a polymer depends on its nature and formulation there are many different behaviors, and hence many different models have been proposed [15,16]. Nevertheless, the most frequent approach to modeling diffusion of small molecules, such as water molecules, through a polymer bulk is to consider Fick’s second law applied to simple single-free-phase diffusion [17,18,19]. Under unsteady state circumstance, Fick’s second law describes the diffusion process as given by Equation (1).

(1) $$\frac{\partial C}{\partial t}=\frac{\partial }{\partial x}\left[D\frac{\partial C}{\partial x}\right]$$

where *C* is the penetrant concentration, *D* a diffusion coefficient and *x* the distance of diffusion. 

Equation (1) stands for concentration change of penetrant at certain element of the system with respect to the time (*t*) for one-dimensional model of linear flow of mass in a solid bonded by two parallel planes.

Assuming D constant in the direction of diffusion Equation (1) can be re-written as:

(2) $$\frac{\partial C}{\partial t}=D\frac{\partial^{2}C}{\partial^{}x^{2}}$$

**Figure 1:**
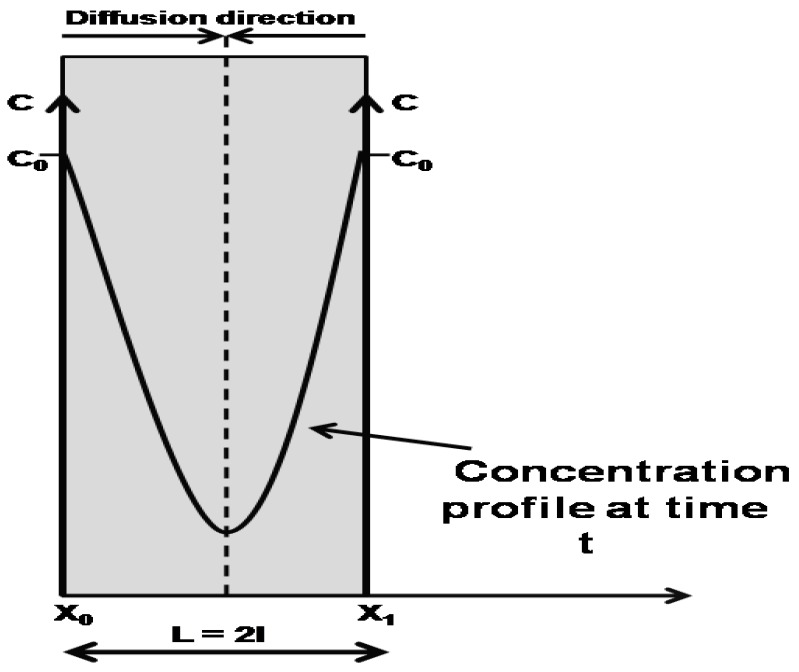
Schematic representation of the concentration profile of penetrant during its diffusion process between two boundaries.

It has been demonstrated by Comyn [16] that for a polymer film of thickness 2*l* immersed into the infinite bath of penetrant, then concentrations, *C_t_*, at any spot within the film at time *t* is given by Equation (3).

(3) $$\frac{C_{t}}{C_{\infty }}=1-\frac{4}{\pi }\sum_{n=0}^{\infty }\frac{{\left(-1\right)}^{n}}{2n+1}\exp\left[\frac{-D{\left(2n+1\right)}^{2}\pi^{2}t}{{4l}^{2}}\right]\cos\frac{\left(2n+1\right)\pi x}{2l}$$

where $C_{\infty }$ is the amount of accumulated penetrant at equilibrium, i.e., the saturation equilibrium concentration within the system. *L* = 2*l* is the distance between two boundaries layers, *x*_0_ and *x*_1_. Simple schematic representation of the concentration profile of the penetrant during the diffusion process between two boundaries is shown in Figure 1.

Integrating Equation (3) over the entire thickness yields Equation (4) giving the mass of sorbed penetrant by the film as a function of time *t*, *M_t_*, and compared with the equilibrium mass, $M_{\infty }$.

(4) $$\frac{M_{t}}{M_{\infty }}=1-\sum_{n=0}^{\infty }\frac{8}{{\left(2n+1\right)}^{2}\pi^{2}}\exp\left[\frac{-D{\left(2n+1\right)}^{2}\pi^{2}t}{{4l}^{2}}\right]$$

For *M_t_*/$M_{\infty }$ ratio ≤ 0.5, Equation (4) can be written as follow:

(5) $$\frac{M_{t}}{M_{\infty }}=1-\frac{8}{\pi^{2}}\sum_{n=0}^{\infty }\frac{1}{{\left(2n+1\right)}^{2}}\exp\left[\frac{-D{\left(2n+1\right)}^{2}\pi^{2}t}{{4l}^{2}}\right]$$

This estimation shows negligible error on the order of 0.1% [20]. 

Equation (5) was simplified by Shen and Springer [19] showing that the initial absorption is given by:

(6) MtM∞=4L(Dtπ)12

where *L* is the film thickness. By plotting the *M_t_*/$M_{\infty }$ ratio as a function of time square root/L, the diffusion constant (D) can be calculated according to the following equation:

(7) $$D=0.0625\;\pi \theta^{2}$$

where θ is the initial slope of the curve in Fick’s plot [21,22].

**Figure 2:**
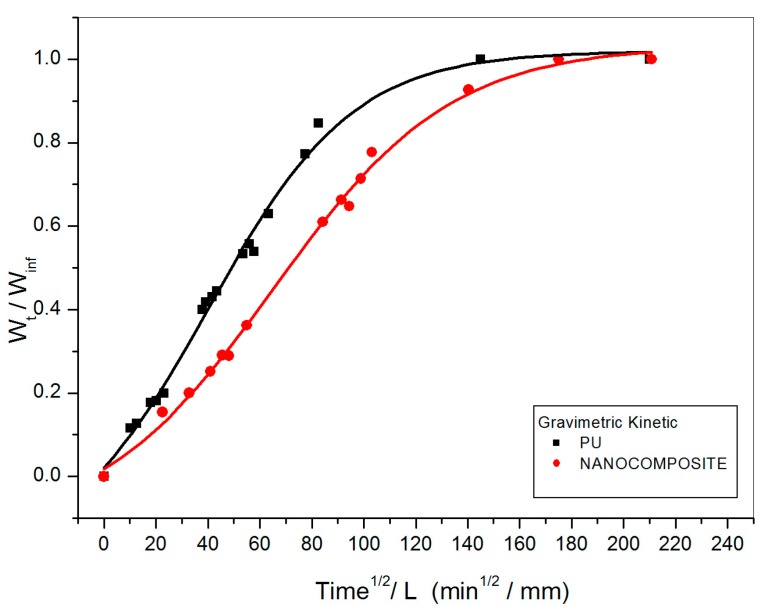
Curves of weight gain versus time for plain poly (carbonate urethane) and TiO_2_/poly (carbonate urethane) nanocomposite systems.

**Figure 3:**
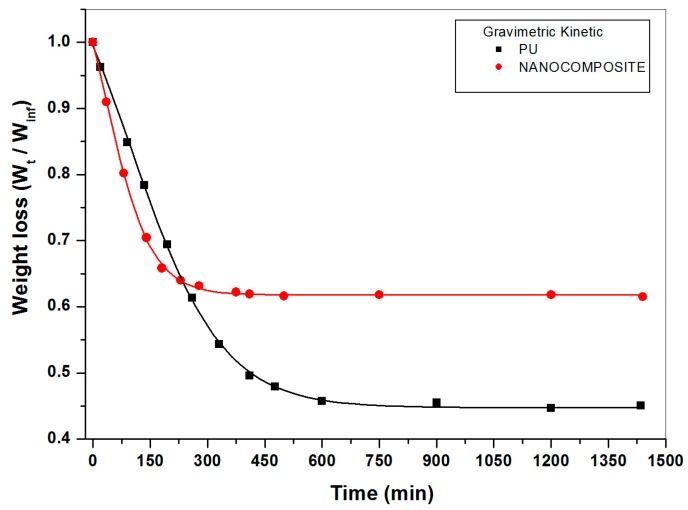
Curves of weight loss versus time for plain poly (carbonate urethane) and TiO_2_/poly (carbonate urethane) nanocomposite systems.

The isothermal sorption curves at 20 °C achieved by gravimetric measurements, shown by both plain poly (carbonate urethane) and nanocomposite wet samples, are typical Fickian diffusion diagrams; i.e., displaying a pronounced linear region in the early stages of the process, afterwards approaching the plateau with a downward concavity (see Figure 2). In Figure 3 the loss in weight due to water desorption as a function of time for the plain poly (carbonate urethane) and nanocomposite wet_I_ samples, simulating a second type of environment such materials could be exploited in, is reported. Such samples were immersed in a deionized water bath at 20 °C until they absorbed a water content constant in the time.

As clearly shown in Figure 2 and Figure 3 a very different behavior is exhibited by the two systems under investigation. The Fickian diffusion coefficients at 20 °C, calculated from the initial slopes of the gravimetric kinetic curves constructed for plain poly (carbonate urethane) and its nanocomposite, are reported in Table 1. In such a table the percentages (wt. %) of water respectively adsorbed at equilibrium and saturation by the two systems are also compared. It should be underlined that irrespective of the environmental conditions set up, the diffusion coefficients calculated for the plain poly (carbonate urethane) are approximately twice as that calculated for the nanocomposite material. As a matter of fact, the overall amount of absorbed water by the nanocomposite is considerably lower than that absorbed by the plain poly (carbonate urethane); the extent of such a lowering increasing strongly at saturation. Notwithstanding that mass transport in a nanocomposite system is heterogeneous, D value representing an average rate over a macro-volume, such results prove that the addition of 1% (wt. %) of TiO_2_ nanoparticles imparts water repellency properties to the PU matrix; i.e., coatings consisting of TiO_2_/poly (carbonate urethane) nanocomposite protect substrates against exposure/ penetration of water and degradation agents conveyed by water.

### 2.2. FT-NIR Measurements

**Figure 4:**
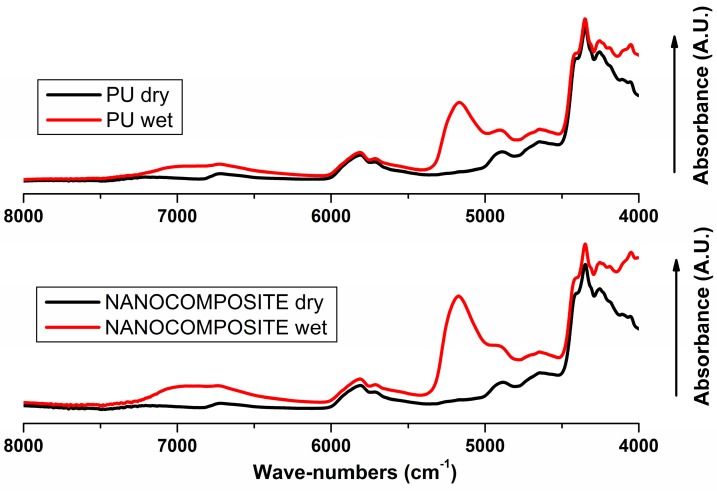
FT-NIR transmission spectra in the wave-number range 8000–4000 cm^−1^ for dry and wet samples of plain poly (carbonate urethane) and TiO_2_/poly (carbonate urethane) nanocomposite.

In Figure 4 the absorbance FT-NIR spectra shown by dry and wet film samples of plain poly (carbonate urethane) and its nanocomposite are respectively reported. Frequencies and assignments of the main absorption bands of the poly (carbonate urethane) phase were reported in a previous work [11], the characteristic absorptions peaks of the plain PU remaining unchanged in presence of the TiO_2_ nanoparticles.

**Figure 5:**
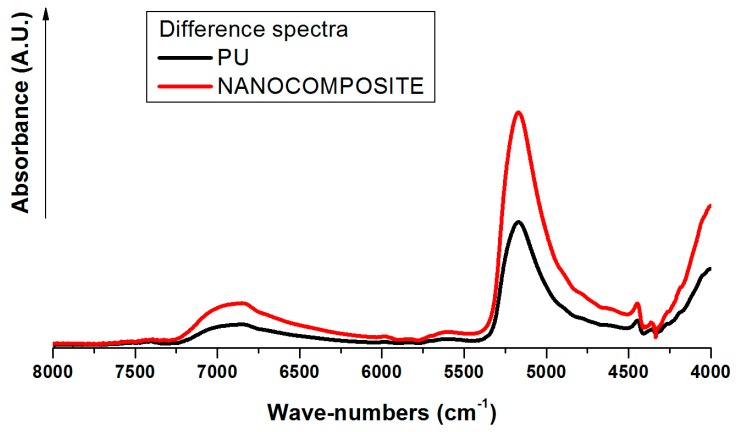
FT-NIR absorbed water spectra for plain poly (carbonate urethane) and TiO_2_/ poly (carbonate urethane) nanocomposite.

A comparison of spectra between dry and wet samples reveal a characteristic peak for absorbed water at 5171 cm^−1^, which is to be assigned to the combination of asymmetric stretching (ν_as_) and in-plane deformation (δ) of water that occurred at 3755 cm^−1^ and 1595 cm^−1^ in the vapor phase spectrum [23]. The 5171 cm^−1^ peak, reasonably resolved, was found appropriate for kinetic studies being free from interference by PU phase and showing a change in intensity strong enough to assess quantitatively the water content in each sample. The absorbed water spectra obtained by difference spectroscopy method [24] representing ν + δ combination peaks, for plain poly (carbonate urethane) and its nanocomposite are shown in Figure 5. As shown, for both the systems under investigation, the similar profile indicating the presence of different water species is observed. It was found that the normalized absorbance of the water band is considerably higher in plain PU than in nanocomposite suggesting that there is a higher amount of equilibrium water uptake in the PU system, in agreement with the results shown by the gravimetric analysis. Moreover, another multicomponent band for water occurs around 6900 cm^−1^ resulting from the combination of ν_as_ and ν_s_ fundamentals. This profile is superimposed onto a much stronger absorption due to the first O–H overtone of the hydroxyl group within the PU matrix producing only a slight increase in the intensity and breath of the band in the 7500–6100 cm^−1^ range. 

Spectroscopic monitoring of the absorbance of the (ν_as_) + (δ) peak representing the overall water diffusion process for the poly (carbonate urethane), without and with the TiO_2_ nanoparticles, was carried out. Time-resolved Fourier Transform Infrared Spectroscopy (FTIR) measurements were performed at different time and the spectrum of water adsorbed was compared with that shown by the dried sample.

**Figure 6:**
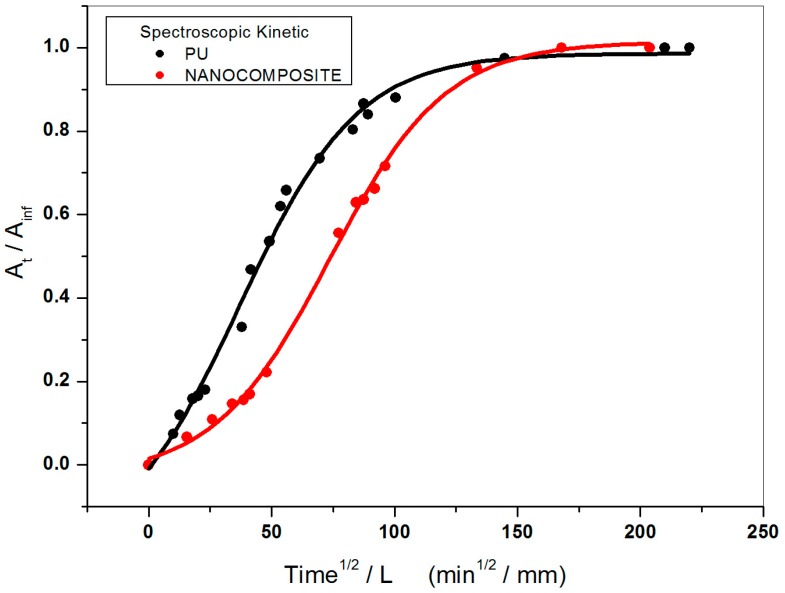
Fick’s curves plotted by spectral data for plain poly (carbonate urethane) and TiO_2_/poly (carbonate urethane) nanocomposite systems.

**Figure 7:**
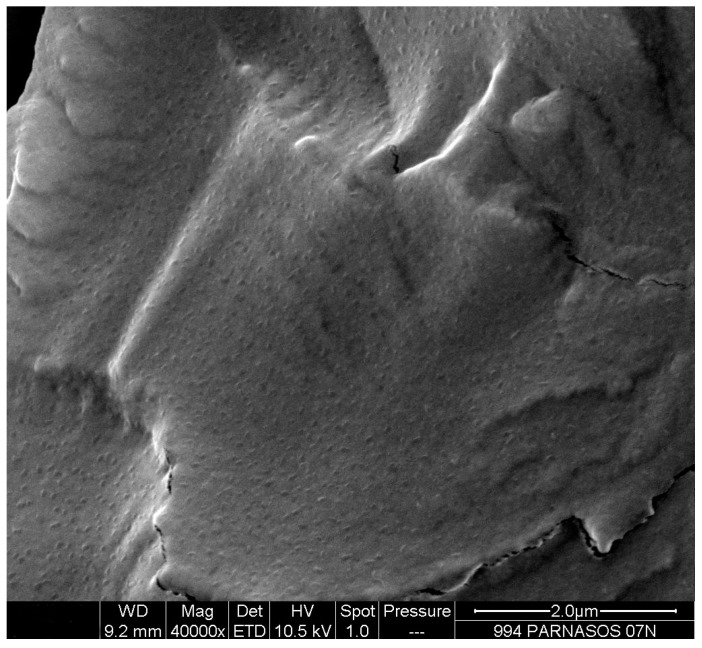
FESEM micrograph of cryogenical fracture surface of TiO_2_/poly (carbonate urethane) nanocomposite film sample.

Suppressing the interference of swelling of the samples during the process of diffusion it is possible to calculate the absolute parameters of diffusion using the equation of Fick as follows [18,19,20,21,22]:

(8) $$\frac{A_{t}-A_{o}}{A_{\infty }-A_{o}}=\frac{C_{t}-C_{o}}{C_{\infty }-C_{o}}=\frac{M_{t}}{M_{\infty }}$$

where *C*_0_, *C_t_*, *C_∞_* represent the concentration of water into sample at time 0, *t*, ∞ at equilibrium. Therefore *C*_0_ *− C_t_ = M_t_* and *C*_0_ *− C_∞_ = M_∞_* represent the mass of water absorbed from the sample at time *t* and at equilibrium respectively. The Fick’s plot obtained from the spectral data is shown in Figure 6. A calibration plot of the recorded absorbance areas normalized for the sample thickness (reduced absorbance) against the content of adsorbed water in milligrams was constructed [24,25,26]. The values of the water diffusion coefficients spectroscopically achieved are reported in Table 1. As expected, at equilibrium, in presence of TiO_2_ nanoparticles the material is confirmed to be comparatively characterized by a lower diffusion coefficient. The finding that D values spectroscopically achieved approach closely D values gravimetrically evaluated (see Table 1) demonstrate, for the systems under investigation, the reliability of FT-NIR way in following the process of water diffusion. It is to be reasonably expected that nanocomposite improved barrier property, observed as a reduction in water uptake, is strongly affected by physico-chemical properties of TiO_2_/PU film such as higher availability of hydrophilic active sites for hydrogen bonding, TiO_2_ mode and state of dispersion, particle size, and morphology, etc. Scanning Electron Microscopy (SEM) analysis of cryogenic fracture surfaces of nanocomposite film shows that the TiO_2_ nanoparticles are homogeneously dispersed and uniformly distributed without significant particle-particle aggregation (see Figure 7); the presence of nanoparticles small clusters resulting in preferential penetrant pathways for water transport [27]. Tortuosity effects of the transport path along with effects of the nanoparticles on PU free volume properties are to be taken also into account.

### 2.3. FT-NIR Curve-Fitting Analysis in the 5400–4600 cm^−1^ Wave-Number Range

Water molecules are well known to dissociate and/or molecularly adsorbed on TiO_2_ surfaces [28,29,30,31,32,33,34]; water behavior being affected by Titanium dioxide surface chemistry and geometry [35,36,37]. Hence, to enhance information on the diffusion process of water through the plain PU and its nanocomposite a curve-fitting analysis in the 5400–4600 cm^−1^ range was performed through PerkinElmer IR Data Manager (IRDM) software (Perkin-Elmer, Beaconsfield, UK). The related deconvolution data are reported in Table 2; the *χ^2^* values, representing the goodness of curve-fitting analysis performed, were 0.049 and 0.045 for plain PU and its nanocomposite respectively. 

As reported in Table 2, for both the systems, a three-water component spectrum is found. Such a finding can be interpreted in terms of a simplified association model, whereby three different water species can be spectroscopically distinguished, on the basis of the strength and the number of H-bonding interactions formed by water with proton accepting groups. In particular, the peak at the higher frequency (5212 cm^−1^) corresponds to those water molecules in which the hydrogens do not form any interaction of the H-bonding type with the systems under investigation. This is not to say that these water species are to be regarded as completely detached from the surrounding polymer chain. Weaker polymer-penetrant interactions undetectable by vibrational spectroscopy, such as dipole-dipole and charge transfer, may still exist. Such kind of water is mobile being localized into excess free volume elements (microvoids and other morphological defects). The component at 5113 cm^−1^ arises from water molecules forming a single H-bonding interaction, whereas the broad component centered at 4931 cm^−1^ originates from water species having both the hydrogens involved in H-bonding with proton acceptor groups. This species may correspond both to single penetrant molecules bridged to two adjacent proton acceptors and to self-associated water in molecular clusters. In the plain PU matrix role of proton acceptor can be most probably played by free carbonyl groups, whether they are in hard or soft segments (i.e., both urethane and carbonate), according to the extent of soft and hard phase mixing considering that –NH groups in urethane linkage are able to form hydrogen bonds with urethane carbonyl and carbonate carbonyl [38]. In the nanocomposite, additional strong proton acceptors are the oxygen atoms in TiO_2_ molecules; the O–H bond being much stronger and more covalent than the O–Ti bond. At the sorption equilibrium, the ratio between the relative fractions of not bonded and bonded water, as calculated by the areas of the absorbance peaks reported in Table 2 for plain poly (carbonate urethane) and nanocomposite, was estimated 0.53 and 0.50 respectively. This finding indicates that the presence of the TiO_2_ nanoparticles reduces the overall amount of absorbed water affecting the fraction of absorbed water molecularly bound.

**Figure 8:**
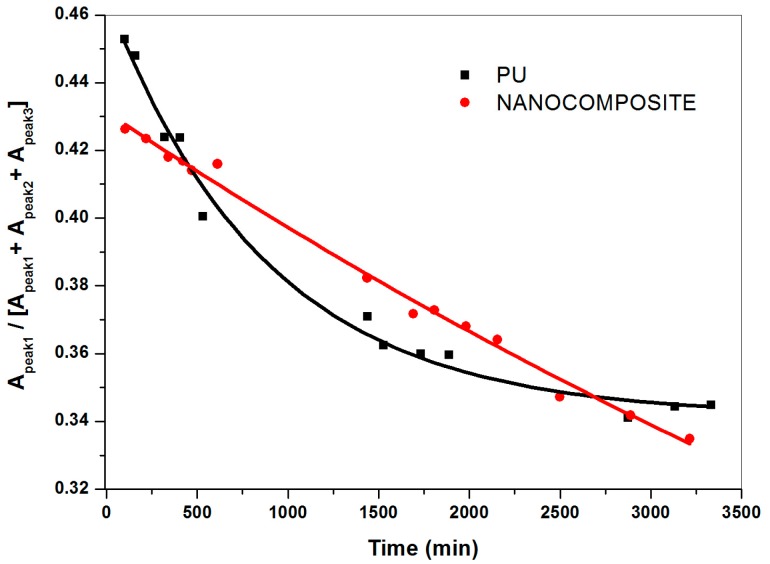
Relative fraction of not bonded water for plain (carbonate urethane) and TiO_2_/poly (carbonate urethane) nanocomposite against the time.

**Figure 9:**
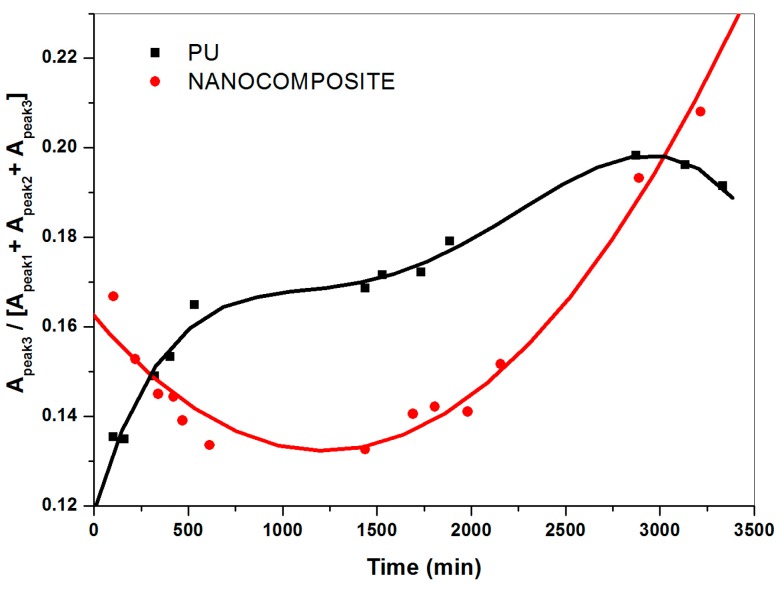
Relative fraction of strongly interacting water for plain (carbonate urethane) and TiO_2_/poly (carbonate urethane) nanocomposite against the time.

**Figure 10:**
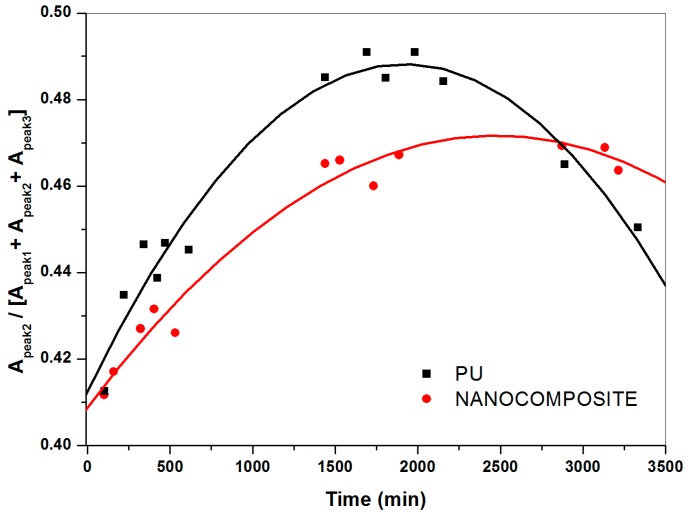
Relative fraction of weakly interacting water for plain (carbonate urethane) and TiO_2_/poly (carbonate urethane) nanocomposite against the time.

The ratio of the area of the individual component peaks to the total absorbance area for the water spectra collected, representing the relative contributions at sorption equilibrium of not bonded, weakly and strongly interacting water, evaluated by curve fitting analysis, are plotted against the time in Figure 8, Figure 9 and Figure 10. Hence, for a given water species, the barrier property exhibited by the nanocomposite system is compared to that shown by the pristine PU system. It is interesting to point out that the addition of TiO_2_ nanoparticles specifically modifies diffusivity, through the PU matrix, of not bonded and strongly bonded water. As a matter of fact, the fraction of not bonded water expected readily desorbed, for the nanocomposite system decreases following a linear trend (see Figure 8).

In presence of TiO_2_ nanoparticles, for the content investigated at least, the transport of such a kind of water occurs with a constant rate. This finding indicates water tendency to spread perfectly across nanocomposite film surface (high wettability) and/or comparable mean free path of water molecules to pass through the polymer matrix. Regardless, such an effect is beneficial in making surfaces easily washable with a plus of oil absorption resistance; i.e., antigraffiti, antisoiling coatings, etc. What is more, in presence of the TiO_2_ nanoparticles the relative contribution of strongly bonded water as a function of time results in downward concavity points. Conversely, upward concavities points are found for the poly (carbonate urethane)/water system (see Figure 9) thus revealing that the addition of TiO_2_ nanoparticles dramatically affects diffusivity of such a kind of water through the polymer matrix. For the pristine matrix system, the upward concavities points are presumably due to the occurrence of water clustering, contributing to an increase in water solubility according to the free volume theory [39,40,41], and to water molecules forming double hydrogen bonds with two already hydrogen-bonded C=O groups. Puffr and Sebenda [42,43] showed that such water molecules are more firmly bounded than that bridging the gaps between the hydrogen-bonded N–H and C=O groups. By contrast, for the nanocomposite system, water absorption immobilized on specific sites, free volume reduction and tortuosity of diffusion path, which the presence of the inorganic nanoparticles causes, could give an account of downwards concavity points.

As far as the contribution of weakly bonded water, the systems under investigation show similar behavior as a function of time (see Figure 10) suggesting that such species of water molecules could jump from one site to another site, irrespective of the TiO_2_ nanoparticles presence. 

It should be pointed out that the analysis used here provides only a limited insight into the water transport in heterogeneous systems such as a polymer-based nanocomposite. On account of the nanocomposites structural and interactional peculiarities, their diffusion kinetics are rather complicated.

### 2.4. DMTA Analysis

**Figure 11:**
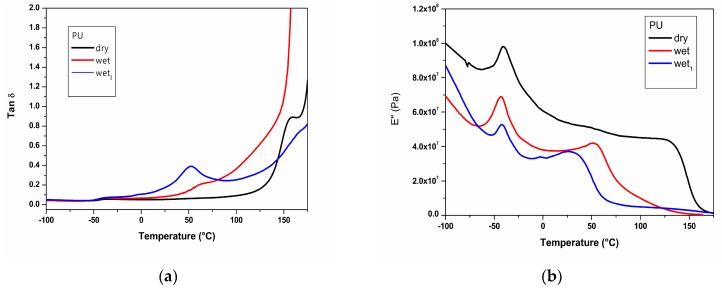

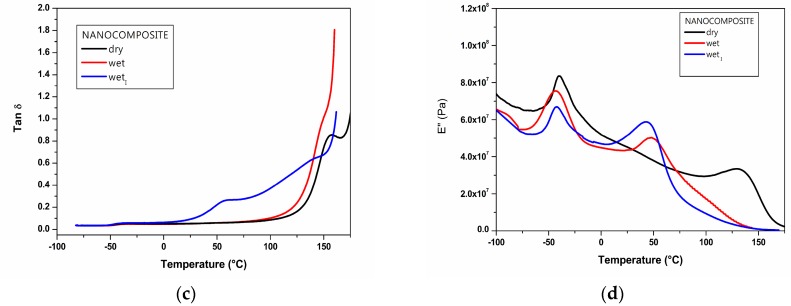
Dynamic-mechanical spectra in terms of loss factor (tan δ) (**a**,**c**) and loss modulus (E″) (**b**,**d**) at 1 Hz for dry, wet and wet_I_ samples of plain poly(carbonate urethane) and TiO_2_/poly (carbonate urethane) nanocomposite.

The dynamic-mechanical spectra in terms of loss factor (tan δ) at 1 Hz for dry, wet and wet_I_ film samples of plain poly(carbonate urethane) and its nanocomposite are shown in Figure 11. In agreement with previous results [11], for both the materials the tan δ plots (Figure 11a,c) reveal the occurrence of two distinct relaxation processes with increasing temperature. Such relaxations are α transition processes corresponding to the glass transition (*Tg*) of poly (carbonate urethane) soft and hard segments respectively. In order to accomplish more accurate data, *Tg* values were defined through the peaks obtained by loss modulus (E″) plot also shown in Figure 11b,d. The *Tg* values for dry, wet and wet_I_ samples of the plain poly (carbonate urethane) and its nanocomposite so achieved are reported in Table 3.

It should be noted that in water absence and in presence of TiO_2_ nanophase, a slight *Tg* increase of hard domains is found. Such a result is in agreement with that achieved through a DMTA multi-frequency analysis revealing that in nanocomposite film samples molecular motions of poly (carbonate urethane) hard segments are restricted by the presence of TiO_2_ nanophase, as higher energy is required for their relaxation [11]. The enhanced *Tg* value could suggest positive PU hard phase-nanoparticles interfacial interactions that reduce cooperative segmental mobility.

**Figure 12:**
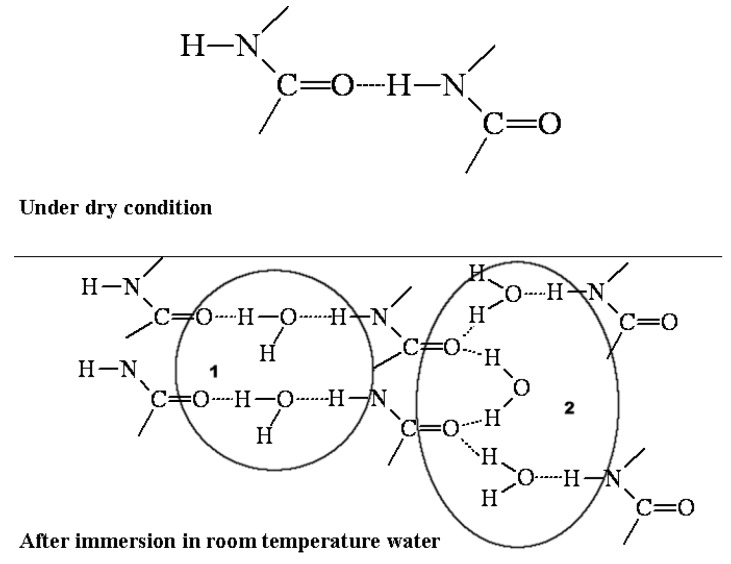
Effects of water on the hydrogen bonding in PUs: (1) weakly bonded water; (2) firmly bonded water.

Polyurethanes are especially prone to moisture-induced plasticization because water molecules can occupy intermolecular hydrogen bonding sites between chains, which would otherwise act as physical crosslinks and restrict chain mobility. Possible water effects on hydrogen bonding are shown by the schematic models reported in Figure 12.

Absorbed water molecules, bridging the gaps between the hydrogen-bonded N–H and C=O groups, weaken hydrogen bonding between N–H and C=O groups. Decrease in hydrogen bonding forces causes decrease in *Tg* together with the function of water as a plasticizer [44,45,46]. Splitting water absorption on the basis of the strength and the number of H-bonding interactions formed by water with PU proton accepting groups as schematically shown in Figure 12, it seems reasonably feasible that free water has negligible effect on the glass transition, while bound water reduces it strongly by weakening the hydrogen bonding between N–H and C=O groups. 

As shown in Table 3, the presence of water decreases the *Tg* values to be ascribed to poly (carbonate urethane) hard phase strongly; this change being thermally reversible upon heating. In contrast, the *Tg* value of poly (carbonate urethane) soft phase is affected scarcely. It has been reported by various researchers that the hydrogen bonding between polymer and inorganic interface can reduce chain mobility and increase *Tg*; i.e., *Tg* confinement effect due to polymer chains confined between nanofillers interfaces [47,48]. Assuming that hydrophilic TiO_2_ nanoparticles take up free volume within PU matrix creating a tortuous path for water molecules and reducing swelling by the water of domains of soft phase, the significant *Tg* reduction observed for the poly (carbonate urethane) hard phase is a combination of reduced hydrogen bonding, water plasticization effect, and polymer-TiO_2_ interactions. The previous two effects could reduce *Tg*, while the last would increase *Tg*.

## 3. Discussion

In order to counteract external degradation of monuments and buildings caused by the atmospheric pollution and meet the demands of cultural heritage with ecological, economic and social aspects, aqueous dispersions of different nanoparticles with photocatalytic capacity were used. Among them, nano-TiO_2_ is one of the most common owing to its versatility and green production, eco-compatibility and low-level impact on the chemical composition of materials. Notwithstanding this, relevant issues are still pending regarding the effectiveness and long-term stability of the coatings “in situ” and the impact of nanoparticles on human health and environment. As an alternative, and with outstanding advantages, the present paper shows that inorganic nanoparticles can be dispersed by means of low impact procedures into polymer matrices suitably selected and that modulation of relevant physical chemical properties such as water-repellency of a protective can be obtained. 

The mechanism through which water diffuses into polymeric materials can be summarized as either infiltration into the free space or specific molecular interactions. The former is controlled by the free space available such as commonly occurring micro-voids and other morphological defects; an increase in the free space should result in an increase of both the water uptake and diffusivity. The diffusion of water by molecular interaction is, on the other hand, controlled by the available hydrogen bond at hydrophilic sites.

For the diffusion of water at room temperature through film samples of TiO_2_/poly (carbonate urethane) nanocomposite gravimetric sorption/desorption tests and FTIR spectroscopic analysis demonstrated that, for the composition investigated at least, the diffusion behavior is Fickian, and substantially linear, in so far as the diffusivity is independent of concentration. The mechanism expected when the diffusion rates are much slower than those of polymer relaxations (Fickian diffusion) can be summarized as follows. At temperatures below *Tg*, the polymer backbone is considered to be in a frozen state, segmental chain motions are drastically reduced, the number of free volume holes is fixed and no hole redistribution is likely. Mass transport is, therefore, assumed to take place via fixed (pre-existing) holes. A penetrant molecule must find its way from hole to hole along pathways involving only minor segmental rearrangements. This means that the diffusivity depends largely on the number of the holes with an appropriate size able to accommodate the diffusing molecule. In the rubbery state above *Tg*, the polymer chains are mobile and the free volume holes show a dynamic variation about size, shape, and position. The penetrant molecules diffuse within the fluctuating interstitial free volume with much greater mobility than in the glassy state.

Moreover, it was shown that the addition of only 1% (wt. %) of hydrophilic TiO_2_ nanoparticles to a poly (carbonate urethane) matrix strongly betters its barrier property. Water absorption in polymer nanocomposites containing impermeable anisotropic domains has been described in several publications. The most common nanocomposites investigated consist of a variety of polymers, both thermoplastic and thermoset, and nanoclay. Transport properties of PUs with soft segments consisting of polycaprolactone/organically modified montmorillonite nanocomposites have been investigated by Tortora et al. [49]. Diffusivity of heterogeneous systems such as polymer nanocomposites is a complex phenomenon. Impermeable domains affect permeability not only by reducing the volume of material available for flow, but also by creating more sinuous pathways according to a tortuous model. Essentially impermeable nanoparticles act as obstacles forcing penetrant molecules to follow longer and complicated routes to diffuse through the material. At the same time, the incorporation of inorganic nano-fillers into the polymer matrix inevitably changes its morphological features and, consequently, its free volume properties. Effects of nanoparticles on polymer free volume to be expected are interfacial regions, interstitial cavities in the filler agglomerates, chain segmental motion immobilization, insufficient chain packaging, changes of the free volume hole size distribution, changes of the crystallinity of the matrix and change of the cross-linking density of the matrix. Which of them become dominant depends primarily on the degree of interaction between the components, the volume fraction of the filler and the geometrical features of the particles. Several studies carried out on reinforced epoxy nanocomposites showed that the maximum water absorption of a polymer system decreased due the presence of nano-filler [50]. Such a phenomenon was generally ascribed to nano-fillers barrier properties together with a tortuous pathway for water molecules to diffuse. The achieved results indicate that the TiO_2_/poly (carbonate urethane) nanocomposite defensive action against penetration of water free from, and bonded through, H-bonding association arises from a balance among TiO_2_ hydrophilicity, tortuosity effects and quality of nanoparticles dispersion and positive inter-facial interactions. Hence, the barrier property of such nanocomposite film is governed by a combination of physico-chemical properties including mode and state of dispersion of the minor component, the interaction between TiO_2_ nanophase and PU matrix, particle size and structure of TiO_2_ nanoparticles, PU morphology and structure, etc. Different analytical techniques, such as Thermo-Gravimetric Analysis–Differential Scanning Calorimetry (TGA-DSC), Field Emission Scanning Electron Microscopy (FESEM), Wide Angle X-ray Scattering (WAXS), DMTA and Attenuated Total Reflectance (ATR)-FTIR were, therefore, applied on both nanocomposite and pristine PU film samples to achieve a thorough characterization [11]. The TiO_2_/poly (carbonate urethane) nanocomposite is a multiphase system in which an inorganic phase with an average size of 31.08 nm was dispersed through sonication. Nanocomposite WAXS intensity profile shows a broad diffraction halo to be ascribed to the amorphous polyurethane phase [6]; no Bragg reflection can be seen corresponding to both the TiO_2_ crystallographic forms Anatase and Rutile [38]. Such a nanophase gives rise to superficial dissociation and/or adsorption and to specific interactions with the water molecules together with interactions with poly (carbonate urethane) hard segments. In turn, the poly (carbonate urethane) phase itself is to be considered as a two-phase amorphous-amorphous system, in which both hard and soft segments are permeable to the water molecules. The morphology of the hard and soft segments of the plain poly (carbonate urethane) was investigated through a careful examination of –NH and carbonyl peaks of ATR-FTIR spectra. It was found that the most of the amide groups are involved in hydrogen bonding [38]. Work is in progress to investigate effects of the addition of TiO_2_ nanoparticles on PU phase separation by means of ATR-FTIR spectroscopy. 

It is to be underlined that the amorphous structure of the TiO_2_/poly (carbonate urethane) nanocomposite confers material a certain degree of rubber elasticity essential for its applications on items with cultural value. In perspective of our final goal, i.e., showing that treatments based on water dispersions of TiO_2_/poly (carbonate urethane) nanocomposite successfully protect outdoor cultural assets stonework, it is to be pointed out that all the effects achieved by the addition of 1% (wt. %) of TiO_2_ nanoparticles are beneficial to combat both exposure/penetration of water and degradation agents conveyed by water and soiling and graffiti. Moreover, it is worthy to note that the nanocomposite *Tg* values, irrespective of water uptake amount, fulfill requirements for protective coatings. At environmental conditions under which weathering processes are most likely to occur, the PU soft phase remains above its *Tg* in an amorphous rubbery state, balanced by the PU hard phase in a glassy amorphous state below its *Tg*.

## 4. Materials and Methods

The raw materials used in this work are reported as follows: a linear aliphatic poly(carbonate urethane) (trade name Idrocap 994) was prepared by ICAP-SIRA (Parabiaco, Milano, Italy) in water dispersion with neutral pH to allow applications on substrates pH sensitive and organic solvents. The prepolymer mixing process followed was reported in a previous work [11]. The Mw values of the poly(carbonate urethane) so achieved are in the range between 30,000 and 50,000 in Gel Permeation Chromatography (GPC) with standard Polystyrene (PS). Titanium dioxide (TiO_2_) nanoparticles were synthesized and kindly supplied in water dispersion by the research center CE.RI.Col of Colorobbia Italia (Sovigliana, Vinci, Florence, Italy). [11]. TiO_2_ nanoparticles have an average size equal to 31.08 nm by Dynamic Light Scattering (DLS) technique with a polydispersity index of 0.241. All the reactants and solvents were used as received.

Plain poly (carbonate urethane) and nanocomposite film samples 0.60–1.00 mm thick were safely achieved using water-casting at room temperature. The preparation of a TiO_2_/poly (carbonate urethane) nanocomposite containing 1% (wt. %) of TiO_2_ nanoparticles was performed by cold mixing the single components via sonication following the low impact method elsewhere reported [11]. Also, the plain poly (carbonate urethane) was undergone identical sonication process. 

Gravimetric sorption measurements were carried out by the so-called pat-and-weight technique. Film samples 0.60–1.00 mm thick were dried for 3 h at 100 °C under vacuum to achieve complete removal of absorbed water. The total absence of absorbed water was confirmed by means of FT-NIR spectroscopy. A Perkin-Elmer Spectrum 100 spectrophotometer (Perkin-Elmer, Beaconsfield, UK) was used. The instrumental parameters adopted for the FT-NIR monitored tests were as follows: resolution 4 cm^−1^, spectral range 8000–4000 cm^−1^. 

FT-NIR spectra exhibited by the dried nanocomposite and plain poly (carbonate urethane) materials were also taken as a reference for spectral subtraction analysis. 

To deeply investigate the effects related to the presence of TiO_2_ nanoparticles on water absorption and desorption kinetics of the poly (carbonate urethane) matrix the following procedures were carried out. Dried film specimens were introduced in an environmental climatic chamber SU250 Angelantoni Industries S.p.a (Cimacolle, Perugia, Italy) at the temperature of 20 °C and 50% of Relative Humidity (RH) simulating weathering. The samples, hereafter wet samples, were removed from the chamber at certain time intervals, weighted in a high precision analytical balance and FT-NIR transmission spectra were collected simultaneously. The amount of absorbed water was calculated by the weight difference. When the content of water remained invariable in the specimens then the kinetics were stopped.

Dried film samples were also immersed in a deionized water bath thermostatically controlled at 20 °C ± 1 °C until they adsorbed a water content constant in the time. The wet samples so achieved, hereafter wet_I_ samples, were introduced in the chamber SU250 Angelantoni Industries (Angelantoni, Naples, Italy) setting the same conditions of temperature and relative humidity used for weathering simulation. Periodically, the samples were removed, blotted and reweighted, the desorption of water was so monitored. In such a procedure we could not apply FT-NIR technique as the high amounts of water absorbed.

Effects of water diffusion on the visco-elastic behavior of both nanocomposite and plain poly (carbonate urethane) were investigated through dynamic mechanical thermal analysis (DMTA) using a Perkin-Elmer Pyris Diamond DMA apparatus (Perkin-Elmer Italia S.p.A, Monza, Italy). Tests were performed in bending mode, applying a strain of 1%. Single-frequency measurements at 1 Hz were performed at a constant heating rate of 3 °C/min, in the temperature range from −100 °C up to 200 °C.

Mode and state of dispersion of the TiO_2_ nanoparticles into the poly (carbonate urethane) matrix were analyzed by means of a Fei Quanta 200 field emission Environmental Scanning Electron Microscope (ESEM, FEI, Hillsboro, OR, USA) operating in high vacuum mode.

## Figures and Tables

**Table 1:** Water diffusion coefficients (D) calculated for the systems under investigation at equilibrium and saturation.

Sample	D (mm^2^/min)	Thickness (mm)	Absorbed Water (wt. %)
PU _(equilibrium)_	Gravimetric: 1.93 × 10^−5^ Spectroscopic: 2.09 × 10^−5^	0.652	15
Nanocomposite _(equilibrium)_	Gravimetric: 1.04 × 10^−5^ Spectroscopic: 9.22 × 10^−6^	1.037	9.95
PU _(saturation)_	Gravimetric: 2.00 × 10^−4^	1.023	85
Nanocomposite _(saturation)_	Gravimetric: 1.16 × 10^−4^	0.975	52

**Table 2:** Results of the Curve-Fitting Analysis of the Spectra of Water absorbed by plain PU and its nanocomposite.

PU						
**Peak**	**Center (cm^−1^)**	**Height (a.u.)**	**Left (cm^−1^)**	**Right (cm^−1^)**	**Fwhh ^a^ (cm^−1^)**	**Area (a.u.)**
Peak 1	5212	0.346	5500	4900	123	53.6
Peak 2	5113	0.336	5600	4600	171	72.0
Peak 3	4931	0.099	5500	4500	239	29.8
**Nanocomposite**						
Peak 1	5211	0.643	5500	4900	122	98.6
Peak 2	5214	0.630	5600	4600	170	134.5
Peak 3	4931	0.198	5500	4500	246	61.3

^a^ Full width at half-height.

**Table 3:** *Tg* values for dry, wet and wet_I_ samples of plain PU and its nanocomposite.

Sample	*Tg* (°C)	*Tg* (°C)
PU_dry_	130	−40
PU_wet_	49	−43
PU_wetI_	30	−42
Nanocomposite_dry_	132	−40
Nanocomposite_wet_	53	−43
Nanocomposite_wetI_	44	−42
